# Radiation-associated degenerative cardiovascular risks during normal aging and after adverse CV event 10 months post-initial exposure

**DOI:** 10.1093/jrr/rrt201

**Published:** 2014-03

**Authors:** Sharath P. Sasi, Xinhua Yan, Juyong Lee, Hamayak Sisakyan, Joseph Carrozza, David A. Goukassian

**Affiliations:** 1 CardioVascular Research Center, GeneSys Research Institute, Boston, MA, USA; 2 Cardiovascular Division, Steward St Elizabeth's Medical Center, Boston, MA, USA; 3 Tufts University School of Medicine, Boston, MA, USA; 4 Department of Cardiology, Yerevan State Medical University, Yerevan, Armenia

**Keywords:** HZE, iron, proton, low-dose, cardiovascular risks, echocardiography, hemodynamics

## Abstract

Background: During the future exploration-type space missions, astronauts will be exposed to ionizing radiation (IR) for more than 1–2 years. The effect of cosmic IR during and after space flights on the cardiovascular (CV) system is unknown. Therefore, it is important to evaluate space IR effects on the CV system and determine potential post-mission degenerative excess relative risks (ERR) to the heart as a function of normal aging (IR + AGING model) as well as determine whether space IR may affect the processes of recovery after an adverse CV event (i.e. acute myocardial infarct, AMI) during normal aging (IR + AGING + AMI model).

Methods: Nine-month-old C57BL6N male mice were IR once with proton (50 cGy, 1 GeV/n) or (^56^Fe 15 cGy, 1 GeV/n). IR-induced alterations in cardiac function were assessed by echocardiography (ECHO) and hemodynamic measurements (HEMO). AMI was induced by ligation of left anterior descending (LAD) coronary artery 10 months post-IR. Mice were monitored over 28 days post-AMI.

Results: Compared with control, in the IR + AGING study group, left ventricular end-systolic pressure (LVESP) was significantly decreased in both ^1^H- and ^56^Fe-IR (*P* < 0.03, both), suggesting IR-associated decrease in contractile function 10 month post-IRs. However, compared with age-matched control mice (18 months), the LV end-diastolic pressure (LVEDP) was significantly increased (*P* < 0.05) and minimum LV pressure change (d*P*/d*t* min, mmHg/sec) was significantly decreased (*P* < 0.02) in ^1^H-IR but not ^56^Fe-IR mice, suggesting that a single 50 cGy full body ^1^H-IR decreases considerably the relaxation function of the heart 10 months post-AMI. Of note, an increase in LVEDP and a decrease in d*P*/d*t* min are indicators that heart is not pumping blood well and is an early independent prognostic CV risk factor for development of cardiac de-compensation.

In all three IR + AGING + AMI study groups, in average, there was 10–15% mortality up to 3 days post-AMI surgery with ∼90% survival rate in all groups 28 days post-AMI. This is rather very good survival rate for 18- to 20-month-old mice after permanent LAD ligation. In the IR + AGING + AMI study group, the most harmful effects on myocardial recovery 10 months post-IR and 28 days post-AMI were observed in the ^56^Fe-IR group. LVESP was significantly decreased in ^56^Fe-IR vs control and ^1^H-IR mice (*P* < 0.04 and <0.02, respectively). LVEDP was 3-fold higher in ^56^Fe-IR vs ^1^H-IR mice (*P* < 0.004) but was only slightly higher (*P* = n.s.) compared with control mice. However, d*P*/d*t* max and d*P*/d*t* min were significantly decreased in ^56^Fe-IR vs control (*P* < 0.007 and <0.05, respectively) and ^1^H-IR mice (*P* < 0.0004 and <0.0015, respectively), suggesting that ^56^Fe-AMI hearts developed cardiac de-compensation.

Summary: Our data in the IR + AGING study group strongly suggest that 10 months post-IR low-dose high-energy ^1^H-IR but not low-dose HZE (^56^Fe) particle IR affects considerably contractile and relaxation functions during normal aging. Conversely, our data in the IR + AGING + AMI study group at 10 months post-IR taken together with our previously reported data for AMI recovery 3 month after a single 50 cGy ^1^H-IR and 15 cGy ^56^Fe-IR indicate that 3 months and as long as 10 months after a single full-body IR, the ^56^Fe-IR is detrimental, whereas ^1^H-IR does not have negative effects on post-AMI recovery. In fact, single ^1^H-IR, at this dose, was considerably beneficial for post-AMI recovery at 3 months, as well as at 10 months post-IR.

Major conclusions: Our longitudinal 1, 3 and 10 months studies in the IR + AGING and IR + AGING + AMI groups reveal that a single full-body low-dose ^1^H and HZE particle radiation (^56^Fe) have long-lasting negative effect on heart homeostasis during normal aging (predominantly ^56^Fe and at 10 months ^1^H-IR, as well), and present a significant CV risk for recovery after adverse CV event (exclusively ^56^Fe-IR, whereas ^1^H-IR at this dose could beneficial). Further, the divergent effects of low dose ^1^H-IR vs ^56^Fe-IR on heart function during normal aging vs after adverse CV event suggest significantly different biological responses responsible for this ion-dependent dichotomy over 10 months post-IR and necessitate further in-depth studies into underlying molecular mechanisms.

